# P300 amplitude is insensitive to working memory load in schizophrenia

**DOI:** 10.1186/1471-244X-11-29

**Published:** 2011-02-15

**Authors:** Pablo A Gaspar, Sergio Ruiz, Francisco Zamorano, Marcela Altayó, Carolina Pérez, Conrado A Bosman, Francisco Aboitiz

**Affiliations:** 1 Clínica Psiquiátrica Universitaria, Hospital Clínico de la Universidad de Chile, Santiago, Chile; 2 The Nathan S. Kline Institute for Psychiatric Research, Orangeburg, New York, USA; 3 Institute of Medical Psychology and Behavioural Neurobiology, Tübingen, Germany; 4 Graduate School of Neural and Behavioural Sciences, International Max Planck Research School. Tübigen, Germany; 5 Centro Interdisciplinario de Neurociencia, Departamento de Psiquiatría, Escuela de Medicina, Pontificia Universidad Católica de Chile. Santiago, Chile; 6 Donders Institute for Brain, Cognition and Behaviour, Centre for Cognitive Neuroimaging. Radboud University Nijmegen, The Netherlands

## Abstract

**Background:**

Working memory (WM) tasks usually elicit a P300 ERP component, whose amplitude decreases with increasing WM load. So far, this effect has not been studied in schizophrenics (SZs), a group that is considered to have an aberrant brain connectivity and impairments in WM capacity. The aim of this study was to determine the dependency of the P300 component on WM load in a sample of SZ subjects.

**Methods:**

We recorded 26 subjects (13 SZ patients and their matched controls) with an 80-channel electroencephalogram. Subjects performed an N-back task, a WM paradigm that manipulates the number of items to be stored in memory.

**Results:**

In healthy subjects, P300 amplitude was highest in the low WM load condition, and lowest in both the attentional control condition and the high WM load condition. In contrast, SZs evidenced low P300 amplitude in all conditions. A significant between group difference in P300 amplitude was evidenced only at the low WM load condition (1 -back), being smaller in SZs.

**Conclusions:**

SZ subjects display a lower than normal P300 amplitude, which does not vary as a function of memory load. These results are consistent with a general impairment in WM capacity in these patients.

## Background

Working memory (WM) refers to a set of cognitive processes that actively hold and manipulate information in the brain for subsequent behavior in the short term [1]. Event Related Potential (ERP) waveforms have been proposed to be markers of cognitive demands during the execution of WM tasks in both normal [2] and schizophrenic (SZ) subjects [3]. More specifically, the P300 component is considered to reflect the activation of widespread fronto-parietal networks, possibly including the anterior cingulate cortex [4,5], involved in attentional and mnemonic processing resources. In normal subjects, this potential has been observed to decrease its amplitude with increasing memory load in WM tasks [6].

SZ is characterized by strong impairments in multiple attentional and WM processes, which have been proposed to be among the core cognitive deficits in this condition [7,8]. Verbal and spatial WM dysfunctions have been consistently reported in first episode patients as well as in groups of non-psychotic persons at familial high risk to develop SZ [9,10]. Thus, the study of WM mechanisms and their impairments in SZ might contribute to unveil some of the underlying affected mechanisms in this disease. Specifically, our aim is to analyze the pattern of activation, as seen in the P300 amplitude, of the SZ brain as a function of increasing WM load.

Although alterations in P300 amplitude have been consistently reported in SZ [11,12], to the best of our knowledge there are no studies that test the P300 pattern at high WM load conditions in these patients. Herein, we compared the behavioral performance and the elicited P300 component under the execution of a verbal N-back task at different WM loads (0-back to 2-back) in a sample of paranoid SZ patients and matched controls.

## Methods

### Participants

Thirteen chronic SZ (paranoid type) outpatients were recruited from the mental health service of the *Pontificia Universidad Católica de Chile*. Two psychiatrists confirmed the diagnosis of SZ (according to the DSM-IV-TR clinical version). Patients were matched by sex, age and socioeconomic status with 13 healthy subjects (HS). All subjects in this study were right handed and had normal or corrected-to-normal vision. Every subject underwent a medical and a psychiatric interview, which included the Mini international neuropsychiatry interview 5.0 (M.I.N.I plus). Demographic data were recollected and a local-validated structured socioeconomic scale was performed. A trained psychologist performed an IQ scale (WAIS) to both patients and controls. Severity assessment of SZ patients was determined with the positive and negative syndrome scale (PANSS) for SZ and the Clinical Global Impression Scale (CGI-S) [13]. Exclusion criteria for this study were any current or past psychiatric diagnosis (excluding schizoaffective disorder diagnosis in the SZ group), substance abuse/dependence, the use of benzodiazepines or anticonvulsive drugs, mental retardation, a clinically significant medical illness or any history of neurological disease. We also excluded any control with family records of SZ, psychosis or bipolar disease. Table 1 shows all relevant clinical and demographic data for patients and controls. Clinical rate evaluation (PANSS and WAIS) was performed no more than 1 month since the EEG recording.

**Table 1:** Socio-demographic variables.

Variable	Healthy Controls	Schizophrenic Patients
		
	n = 13	n = 13
Age (y), mean (SD)	31.2 (12.1)	30.3 (10.6)
Sex, n (%) male	9 (69.2)	10 (76.9)
Symptom severity scale (PANSS)		
		
(30-210), score (SD)		
Negative scale	-	20.8 (6.59)
Positive scale	-	14.5 (4.84)
General psychopathology	-	36.4 (12.1)
Total score	-	71.7 (20)
Clinical rated CGI severity score (CGI-s)		
		
(1-7), score (SD)	-	4(6.4)
Socio economic scale, level, n (%)		
		
High	4 (30.8)	3 (23.1)
Medium	9 (69.2)	10 (76.9)
Low	0	0
Years of treatment		
		
mean (SD)	-	12.9 (11.4)
Chlorpromazine equivalent		
		
doses mg/day	-	419.2 (186.5)
Antipsychotic treatment n (%)		
		
First generation	-	12 (92.3)
Second generation	-	1 (7)

Socio economic scale includes: education, employment status, family economic status and graduate living conditions.

This protocol was approved for the ethics committee of the *Pontificia Universidad Católica de Chile*. Every subject signed an informed consent. In case of SZ patients, this consent was also signed by a well-informed relative.

### Task and stimulation procedures

**Figure 1:**
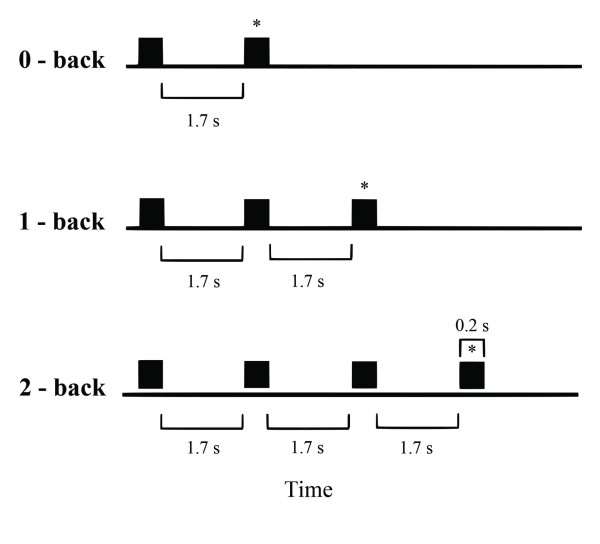
**N-back task timeline**. Trial time sequences for 0-, 1- and 2- back conditions. Black squares represent each stimulus in the task. The symbol * shows the target number during each trial. Inter stimulus interval (ISI): 1.7 sec. Stimulus presentation: 0.2 sec.

WM was assessed using an implicit verbal N-back task [6,14], in which subjects were presented a sequence of digit numbers, and had to determine whether the currently displayed stimulus at any given time had been already displayed in the previous presentation (1-back condition, low WM load); or in the second-to-previous presentation (2-back condition, high WM load) (see figure 1). There was also a control condition (0-back), in which the subject had to recognize a specific digit -zero- when it appeared on the screen. Subjects had to distinguish between targets and non-targets, by pressing two buttons localized in a response palette. Reaction times were recorded after pressing the button. A trial was defined by the presentation of one number followed by the subject's motor response. Trials were presented in 3 blocks; each block representing either the control (0- back) or WM conditions (1- and 2- back). Each block consisted in 180 trials with a 1:1 target/no target relation [6]. Stimuli consisted in a 0.2 sec. presentation of a gray digit (size: 2.6 × 5.2 deg at 65 cm. from the face) located in the center of a black background screen in a 21' CRT monitor. Inter stimulus interval (ISI) were 1.7 sec in all the conditions studied. All the subjects used the dominant hand to respond. Stimulus presentation was implemented using the STIM 1.0 software (Compumedics-Neuroscan^®^).

### Data acquisition

Continuous EEG signals were acquired using an 80-channel electroencephalographic system (Neuroscan^® ^EEG Nuamps device). Electrodes were placed using a 10/20 extended QuikCap system (Neuroscan^® ^Inc). References were placed at vertex by default, but were subsequently off-line re-referenced to averaged mastoids. Impedance values were kept at 5 KΩ for all electrodes. We used three external flat electrodes to monitor eye movements (two above and below the left eye and one 3 cms. next to the outer canthus of the right eye). Recordings were sampled at 1000 Hz and band-pass filtered between 0.1 and 100 Hz using an on-line amplifier.

### Event Related Potential analyses

Trials with undesired eye movements and eye blink artifacts were eliminated from the analysis using a semi-automatic and manual block rejection procedure. To remove unwanted ERP components, such as the CNV like component evoked in this kind of task [15], we executed an offline digital band-pass filter from 2 to 30 Hz (zero phase shift filter). The continuous EEG was subsequently segmented between 500 ms. previous to the appearance of a target stimulus to 800 ms. after stimulus onset. However, the baseline used for the ERP analysis was 200 ms. previous to the appearance of the target stimuli. We included in our analysis only successful trials (defined as match stimuli). Individual segments were excluded if the absolute voltage of each channel was higher than 80 μV. In each subject, successful, artifact-free trials were averaged in each task (0 -1- and 2- back tasks) to obtain the corresponding ERP waves [16]. Subjects with less than 30 epochs to average for each task were excluded. The number of trials (mean and error rate) of each condition (0-, 1- and 2-back) and group (HS and SZ) were: Control group: 0- back: 46.5 (4.2), 1- back: 47.3 (3.9), 2- back: 43.3 (4.5); SZ group: 0- back: 50.5 (4.8), 1- back: 41.8 (4.5), 2- back: 38.6 (4.5). Finally, we calculated a group average over the ERPs obtained across subjects for visualization purposes. All these analyses were made using Scan 4.3 (Compumedics-Neuroscan^®^), Matlab 7.0 software (The Mathworks Inc.) and the EEGLAB 4.5 toolbox.

### Statistical analyses

The behavioral effects of WM load (0-, 1- and 2- back tasks) and group (patients and controls) were statistically evaluated using a repeated measures analysis (ANOVA general linear model). Hit rate (HR) was defined as percentage of correct responses, while reaction times (RTs) were defined as the first response of the subject 200 ms. after the appearance of target stimulus. Greenhouse-Geisser and Bonferroni methods were used to correct compound symmetry violations in the ANOVAs. Post Hoc analysis and main-effect comparisons were adjusted using the Bonferroni correction. Uncorrected DFs were reported for each F statistics. Statistical analysis between conditions (0-, 1- and 2-back) and groups (HS and SZ) was performed using non-parametric Mann-Whitney tests and confirmed by cluster permutation procedure [17]. In the latter, the cluster-level test statistics is defined by pooling the z scores of neighboring electrodes showing the same effect (pooled z scores >1.96) in a given time window of interest. The type I error rate for the complete set of electrodes was managed by evaluating the cluster-level test statistics under the randomization null distribution of the maximum cluster-level statistics. This was obtained by randomly permuting the data between conditions and between groups. By creating a distribution from 100 random sets of permutation, statistical significance (p < 0.05) was estimated as the proportion of elements in the randomization null distribution exceeding the observed maximum-cluster level test statistics.

Additionally, we performed an analysis of regions of interest (ROI) by averaging fifteen neighboring electrodes for each one [18]. The 5 resulting ROIs were labeled as follows: central midline (CMROI), frontal right (FR-ROI), frontal left (FL-ROI), parietal right (PR-ROI) and parietal left (PL-ROI). Using these measures, two different analyses were performed. First, we assessed whether changes in P300 amplitude/latency related to WM load, by comparing ERPs from low WM load (1-back) and high WM load (2- back) conditions within each group (SZ and HS). Second, to compare SZ group with controls, we compared the averaged ERP values for each condition between both groups. In addition, we subtracted the high WM load condition from the low WM condition to each subject, and resulting differences were compared across groups.

## Results

### Behavioral responses

**Figure 2:**
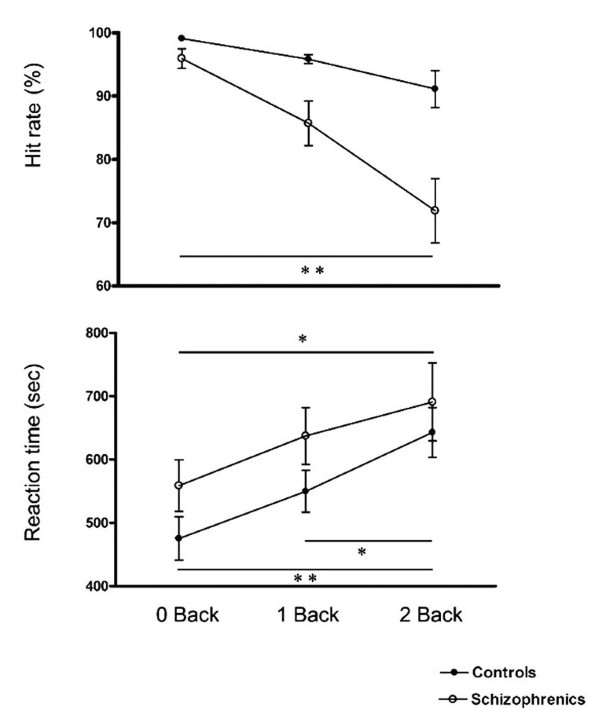
**Task behavioral performance**. Lines graph representing Hit rate (upper panels, %+S.E.M.) and reaction time (lower panels, ms +S.E.M.) for SZ patients (empty circle) and HS (filled circle) in all the conditions (0-,1- and 2 back). * Symbol represent significant: p < 0.05; and ** represent p < 0.01.

In each WM condition, HR percentages were more than 90% and 70% in control and SZ patients, respectively. As expected, in both groups we found increments in RTs and decrements of HRs due to the increase in WM load. In the control group, only the RT differences between 0- to 2- and 1- to 2- back tasks were significant. In the SZ group differences were significant from 0- to 2- back in both HT and RT (figure 2). Between groups, SZ patients made more errors and had longer reaction times than HS in each condition (figure 2). These differences were significant in both 1-back and 2-back tasks, for HR [F(2.1) = 16.7, p < .001] as well as in the RT task [F(1.5) = 4.7, p = .028].

### EEG data analyses

**Figure 3:**
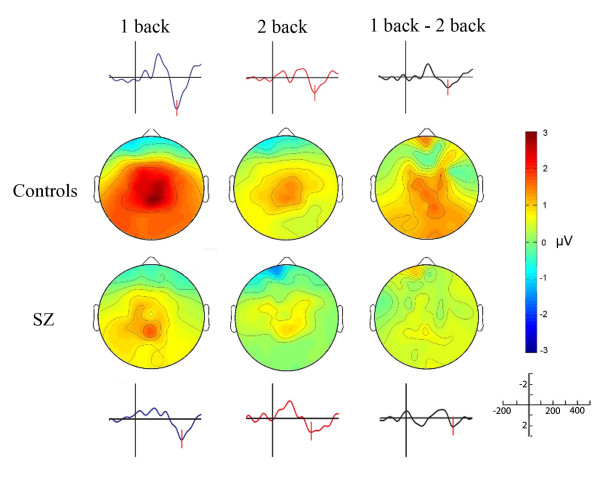
**Grand averages and topographical distribution of the evoked P300 potentials elicited by 1- and 2- back tasks in the CM-ROI**. The third column represents the subtraction of the P300 amplitudes at 1- and 2- back conditions for HS and SZ group. The differences were significant for controls (p <0.05), but not for SZ group (p > 0.05). Color bar indicates amplitude (uV).

Within-group analyses: The control group showed a significant decrease of P300 peak amplitude from 1- to 2-back (p = 0.018, Mann- Whitney test) and from 0- to 2- back condition (p = 0.023, Mann-Whitney test). On the contrary, SZ patients did not show a significant difference in P300 peak amplitude among any condition studied (from 0- to 1- back, p value = 0,56; 0- to 2- back p value = 0,34; and 1- to 2- back p value = 0,086). Besides, we did not find significant differences in P300 latency between 1- and 2- back tasks inside each group (Table 2 and figure 3).

**Table 2:** Statistical analysis of the P300 component in the CM- ROI.

	P300 amplitude (μV)				P300 latency (ms.)			
	**mean (SD)**				**mean (SD)**			
**Condition**	**HS**	**SZ**	**Dif**	**p value**	**HS**	**SZ**	**Dif**	**p value**
	
0 back	3.17 (2.7)	2.80 (1.4)	1.37	0.189	320 (49.9)	331 (47.7)	-11	0.170
1 back	3.29 (1.9)	1.43 (1.0)	1.85	0.019*	330 (29.0)	337 (41.3)	-7	0.869
2 back	2.24 (1.4)	1.36 (0.9)	0.87	0.076	330 (42.7)	332 (40.7)	-14	0.856
	p value				p value			
						
1-2 back	0.023	0.086			0.87	0.84		

Comparison of amplitude and latency measures among SZ group and HS. * p value < 0.05 (Mann-Whitney test).

Between-group analyses: The mean P300 peak amplitude was higher in the control group for every WM condition, but this difference was significant only in the 1- back task (Mann-Whitney test, p = 0,019, see Table 2 and figure 3). The decrement in P300 amplitude between 1- and 2- back was higher in the control group (p = 0.023; Mann-Whitney test). Finally, there was no difference of P300 latency in the SZ group compared with controls (See Table 2 and figure 3).

## Discussion

Consistent with previous results, behavioural performance during the N-back task is better in HS compared with SZ patients [19]. As expected, differences in hit rate and reaction times between SZ patients and controls became larger with higher WM demands. In addition, our ERP findings are in general consistent with previous proposals suggesting that a diminished P300 component represents a marker of cognitive dysfunction in SZ, possibly representing an endophenotype of the illness [12,20]. As opposed to the control group, in SZ subjects we failed to observe a decreased P300 peak amplitude from low (attentional) to high WM load conditions (See figures 2 and 4). Interestingly, the between group comparison of each condition studied (0-back, 1-back and 2-back) displayed a significant difference only at the low WM load condition (1-back).

Functional neuroimaging [21,22] and electrophysiological approaches [23] suggest that SZ patients show important dysfunctions in the prefrontal cortex and in widespread cortical networks [24], which is consistent with our findings of a systematically reduced P300 in these patients. The cognitive interpretation of the P300 has been widely debated, and has different interpretations depending on the author and the specific cognitive task that is assessed. Furthermore, it is likely that the P300 represents a family of related potentials related to different aspects of WM processing [5,25]. A current model states that this potential reflects attentional capacity invested in the categorization of task relevant events [4] . More specifically, this author proposes that the neural network underlying the P300 participates in the comparison of the stimulus attributes with a mental representation of the target, a function that is dependent on attention, working memory load, and task difficulty. The difficulty of our task may partially account for the diminished P300 in our SZ subjects, as they showed a lower hit rate than controls (70% vs. 90%, respectively). In addition, in these patients a reduced P300 may also reflect either a deficit in WM or attentional capacity, or an inability to correctly compare the received sensory percept with the short-term memory representation that is active at the time.

We did not find significant differences of P300 latency between groups (HS and SZ). The observation of a prolonged latency in SZ patients is controversial. A few studies have described a P300 latency prolongation in SZs and their siblings, suggesting a slower stimulus processing [26,27], but other reports failed to find this effect [20].

Finally, some limitations should be considered for the right interpretation of our results. Although our P300 amplitude measures were consistent among subjects, the size of the sample in this study (n = 26) is rather small. The absence of a) P300 latency alterations in our results and b) a correlation between some psychopathological measures (PANSS and CGI) and P300 amplitude might be explained by the small size of subjects. Larger samples of patients will be needed to confirm the possible contribution of these and other clinical factors (such as sex or progression of the disease) in these results.

## Conclusions

In this study, we observed differential patterns of P300 in HS and SZ patients, as a function of WM load. More specifically, in the SZ sample there was a notorious invariance of the P300 at different WM loads, which was not different from that elicited by a primarily attentional task (0-back). Our findings point to a general impairment of attentional and WM capacity in these patients.

## Competing interests

The authors declare that they do not have competing interests.

## Authors' contributions

PAG participated in the conception and design of the study, acquisition of the data, analysis and interpretation of the results, and in drafting the manuscript. SR participated in selection of the participants, acquisition of the data, and in drafting the manuscript. FZ and CAB participated in the acquisition of the data, analysis and interpretation of the results and drafting the manuscript. MA and CP helped in the acquisition of the data and selection of the participants. FA participated in the conception and design of the study, interpretation of the results, and in drafting the manuscript.

All authors read, critically revised, and approved the final manuscript.

## Pre-publication history

The pre-publication history for this paper can be accessed here:

http://www.biomedcentral.com/1471-244X/11/29/prepub
